# Learning Curves Associated With Robotic Total Hip Arthroplasty: A Scoping Review

**DOI:** 10.1111/os.70130

**Published:** 2025-07-23

**Authors:** Abith Ganesh Kamath, Saran Singh Gill, Srikar Reddy Namireddy, Matija Krkovic

**Affiliations:** ^1^ Imperial College London London UK; ^2^ Addenbrooke's Hospital Cambridge UK; ^3^ University of Cambridge Cambridge UK

**Keywords:** learning curves, orthopedic surgery, robotics

## Abstract

**Introduction:**

Robotic total hip arthroplasty (rTHA) is gaining widespread adoption, yet the learning curve (LC) associated with its implementation remains uncertain. Understanding LCs is crucial to optimizing training protocols and improving patient outcomes. This scoping review assesses LCs in rTHA by evaluating operative time, leg length discrepancy (LLD), and acetabular component inclination (ACI).

**Methods:**

A systematic search was conducted across PubMed, MEDLINE, Embase, Scopus, and Web of Science following PRISMA guidelines. Studies assessing the LC of rTHA based on operative efficiency, radiographic accuracy, and surgical outcomes were included.

**Results:**

A total of 12 studies were included. Improvements in operative time were observed at a median of 13 cases, ranging from 7 to 35 cases. Both LLD and ACI findings were inconsistent, with little evidence of a LC found in the literature.

**Conclusion:**

This review highlights the learning curve in rTHA, with proficiency improving after early cases. Standardized benchmarks and training models could enhance learning and enable comparisons across robotic systems. Future research should refine proficiency thresholds, assess system differences, and develop structured training for optimal rTHA adoption.

## Graphical abstract

This scoping review evaluates the learning curve in robotic total hip arthroplasty, revealing improvements in operative time after 7–35 cases. However, findings for leg length discrepancy and acetabular component inclination were inconsistent. Standardized training protocols are needed to optimize proficiency and compare robotic systems.

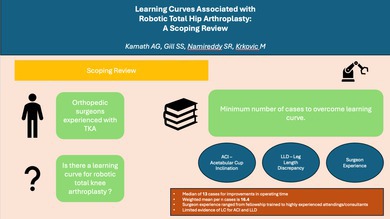

## 1 Introduction

The Centers for Disease Control and Prevention (CDC) estimates that the lifetime risk of developing symptomatic hip OA is 18.5%, with a prevalence of 25.3% respectively [1, 2, 3]. Hip OA is the most common site for severe osteoarthritis, resulting in severe pain and restriction in function [4, 5]. Risk factors include obesity, genetics, lifestyle factors, age, and trauma [6]. It is widely accepted that the risk of hip fractures increases with age, resulting in up to 14.2 million cases per year, globally [7, 8]. The number of NHS‐funded hip replacements per 100,000 population rose substantially from 272.6 and 266.7 in 2002 to 539.7 and 466.3 in 2018 in England and Wales respectively [9]. Consequently, a significant challenge facing orthopedic surgeons is to enhance the efficiency of total hip arthroplasty (THA). This can be achieved by evaluating the learning curve (LC) associated with robotic THA and working on improvements to optimize patient outcomes.

While robotic surgery is now a staple of orthopedic surgery, its usage has been rising exponentially, with the utilization of robotic THA increasing from < 0.1% to 2.1% from 2008 to 2018 [10, 11, 12]. Robotic surgery plays a key role as an adjunct to traditional surgery, with varying levels of efficacy across different fields including cardiothoracic, urology, and gastroenterology [13, 14, 15]. Responses from 304 surgical trainees on behalf of the Association of Surgeons in Training (ASiT) in the UK view robotic surgery as a necessary advancement that will become more common in the future, with 73.4% believing that robotic surgery is important for the future of their desired specialty and 77.2% believing it should be incorporated into formal surgical training [16]. However, the learning curves (LC) associated with common procedures such as THA are still speculative, with uncertainty regarding their impact on surgical outcomes [17]. Given the potential of robotic surgery to transform the surgical field, it is pivotal to quantify and assess LC for various aspects of robotic surgery to improve the quality and sustainability of surgical training overall [18]. Clinical assessments of LC within robotic neurosurgery and otorhinolaryngology demonstrate variations in surgical performance with experience [19, 20, 21]. A study conducted by the Cleveland Clinic observed a significant increase in the adoption of robotic arm‐assisted THA (RA‐THA), projecting that by 2025 approximately 23.9% of THAs will be done with robotic assistance [22]. This growing trend underscores the need for a deeper understanding of the learning curve associated with robotic THA, highlighting the importance of structured training programs to equip the next generation of orthopedic surgeons with the necessary skills and expertise. As such, analyzing the LC in robotic THA remains a critical area of interest.

While prior reviews, such as Soomro et al.'s assessment of LC in robotic surgery more broadly, have addressed the concept at a general level, few have specifically explored LC within robotic THAs [17]. To the best of our knowledge, this is the first scoping review to assess LC in robotic THA, aiming to synthesize current evidence using operative time and procedure specific outcomes including leg length discrepancy (LLD), and acetabular component inclination (ACI), to evaluate the progression of surgical proficiency [17, 23, 24, 25, 26, 27].

## 2 Methodology

### 2.1 Literature Search Strategy

**FIGURE 1:**
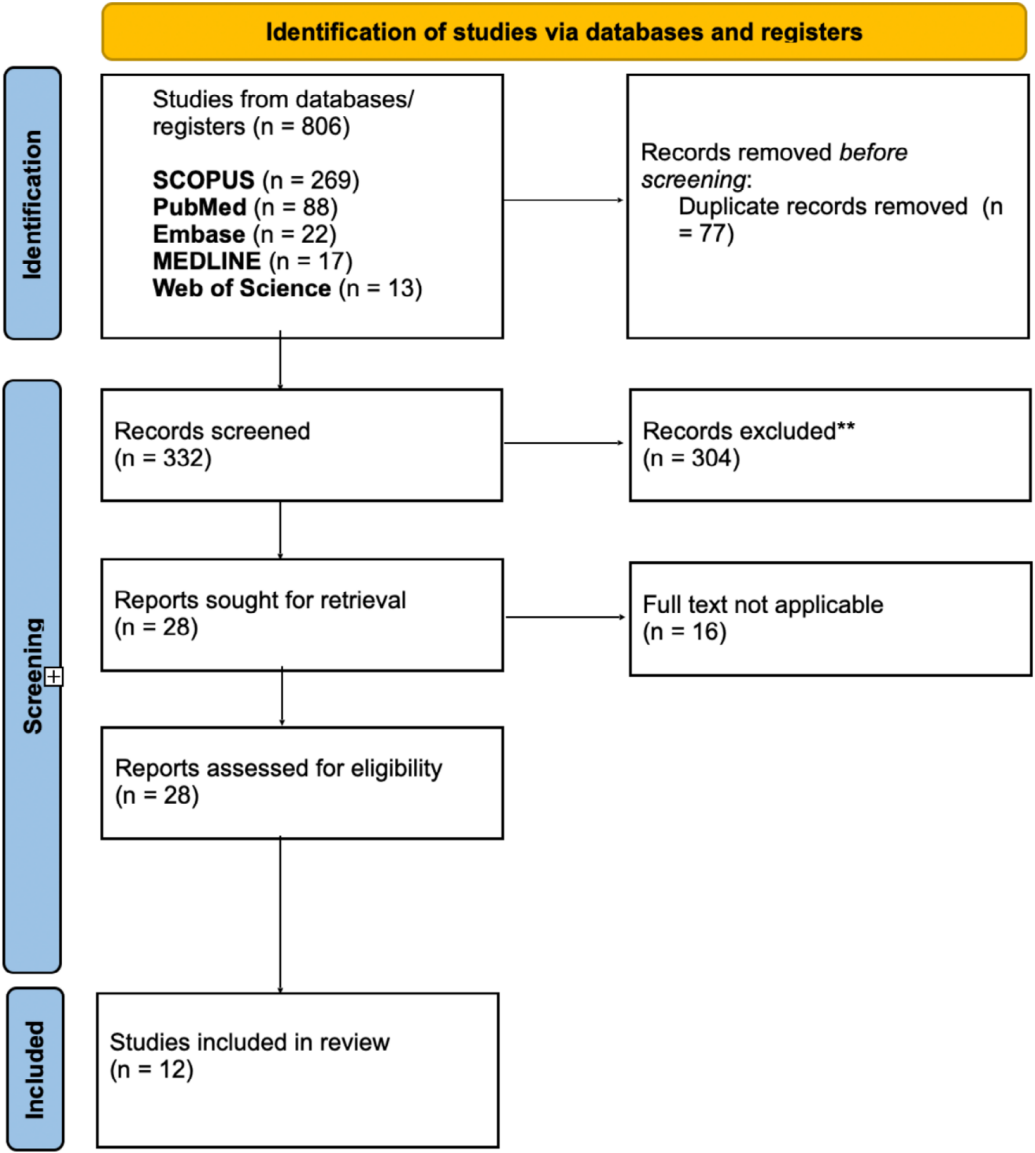
PRISMA.

This scoping review was conducted following the guidelines outlined by the Cochrane Collaboration and the Preferred Reporting Items for Systematic Reviews and Meta‐Analyses Scoping Review Extension(PRISMA‐ScR) [28]. The PRISMA flowchart summarizing the selection process is presented in Figure 1A. A comprehensive literature search was performed on January 15, 2024, across four major databases: PubMed, MEDLINE, Embase, Scopus, and Web of Science. Search strings were developed to address the research question: “What are the learning curves associated with robotic total hip arthroplasty?” The full search strategy is detailed in Table 1.

**TABLE 1:** Search strategy.

Database (Results)	Search terms
PubMed: (88)	(“robotic hip surgery” OR “robotic‐assisted hip surgery” OR “robotic hip arthroplasty” OR “robotic‐assisted hip arthroplasty” OR “robotic total hip arthroplasty” OR “robotic‐assisted total hip arthroplasty” OR “robotic hip replacement” OR “robotic‐assisted hip replacement” OR “robotic hip resurfacing” OR “robotic‐assisted hip resurfacing” OR “robotic periacetabular osteotomy” OR “robotic‐assisted periacetabular osteotomy”) AND (“learning curve” OR “training” OR “proficiency” OR “skill acquisition” OR “competency” OR “experience” OR “performance improvement”)
SCOPUS: (269)	TITLE‐ABS‐KEY ((“robotic hip surgery” OR “robotic‐assisted hip surgery” OR “robotic hip arthroplasty” OR “robotic‐assisted hip arthroplasty” OR “robotic total hip arthroplasty” OR “robotic‐assisted total hip arthroplasty” OR “robotic hip replacement” OR “robotic‐assisted hip replacement” OR “robotic hip resurfacing” OR “robotic‐assisted hip resurfacing” OR “robotic periacetabular osteotomy” OR “robotic‐assisted periacetabular osteotomy”) AND (“learning curve” OR “training” OR “proficiency” OR “skill acquisition” OR “competency” OR “experience” OR “performance improvement”))
OVID embase: (22)	((“robotic hip surgery”.sh. OR “robotic‐assisted hip surgery”.sh. OR “robotic hip arthroplasty”.sh. OR “robotic‐assisted hip arthroplasty”.sh. OR “robotic total hip arthroplasty”.sh. OR “robotic‐assisted total hip arthroplasty”.sh. OR “robotic hip replacement”.sh. OR “robotic‐assisted hip replacement”.sh. OR “robotic hip resurfacing”.sh. OR “robotic‐assisted hip resurfacing”.sh. OR “robotic periacetabular osteotomy”.sh. OR “robotic‐assisted periacetabular osteotomy”.sh.) AND (“learning curve”.sh. OR “training”.sh. OR “proficiency”.sh. OR “skill acquisition”.sh. OR “competency”.sh. OR “experience”.sh. OR “performance improvement”.sh.))
OVID medline: (17)	((“robotic hip surgery”.sh. OR “robotic‐assisted hip surgery”.sh. OR “robotic hip arthroplasty”.sh. OR “robotic‐assisted hip arthroplasty”.sh. OR “robotic total hip arthroplasty”.sh. OR “robotic‐assisted total hip arthroplasty”.sh. OR “robotic hip replacement”.sh. OR “robotic‐assisted hip replacement”.sh. OR “robotic hip resurfacing”.sh. OR “robotic‐assisted hip resurfacing”.sh. OR “robotic periacetabular osteotomy”.sh. OR “robotic‐assisted periacetabular osteotomy”.sh.) AND (“learning curve”.sh. OR “training”.sh. OR “proficiency”.sh. OR “skill acquisition”.sh. OR “competency”.sh. OR “experience”.sh. OR “performance improvement”.sh.))
Web of science: (13)	((“robotic hip surgery” OR “robotic‐assisted hip surgery” OR “robotic hip arthroplasty” OR “robotic‐assisted hip arthroplasty” OR “robotic total hip arthroplasty” OR “robotic‐assisted total hip arthroplasty” OR “robotic hip replacement” OR “robotic‐assisted hip replacement” OR “robotic hip resurfacing” OR “robotic‐assisted hip resurfacing” OR “robotic periacetabular osteotomy” OR “robotic‐assisted periacetabular osteotomy” OR “robotic hip procedures” OR “robotic hip operations”) AND (“learning curve” OR “training” OR “proficiency” OR “skill acquisition” OR “competency” OR “experience” OR “performance improvement” OR “surgical skills” OR “education”))

### 2.2 Inclusion and Exclusion Criteria

**FIGURE 2:**
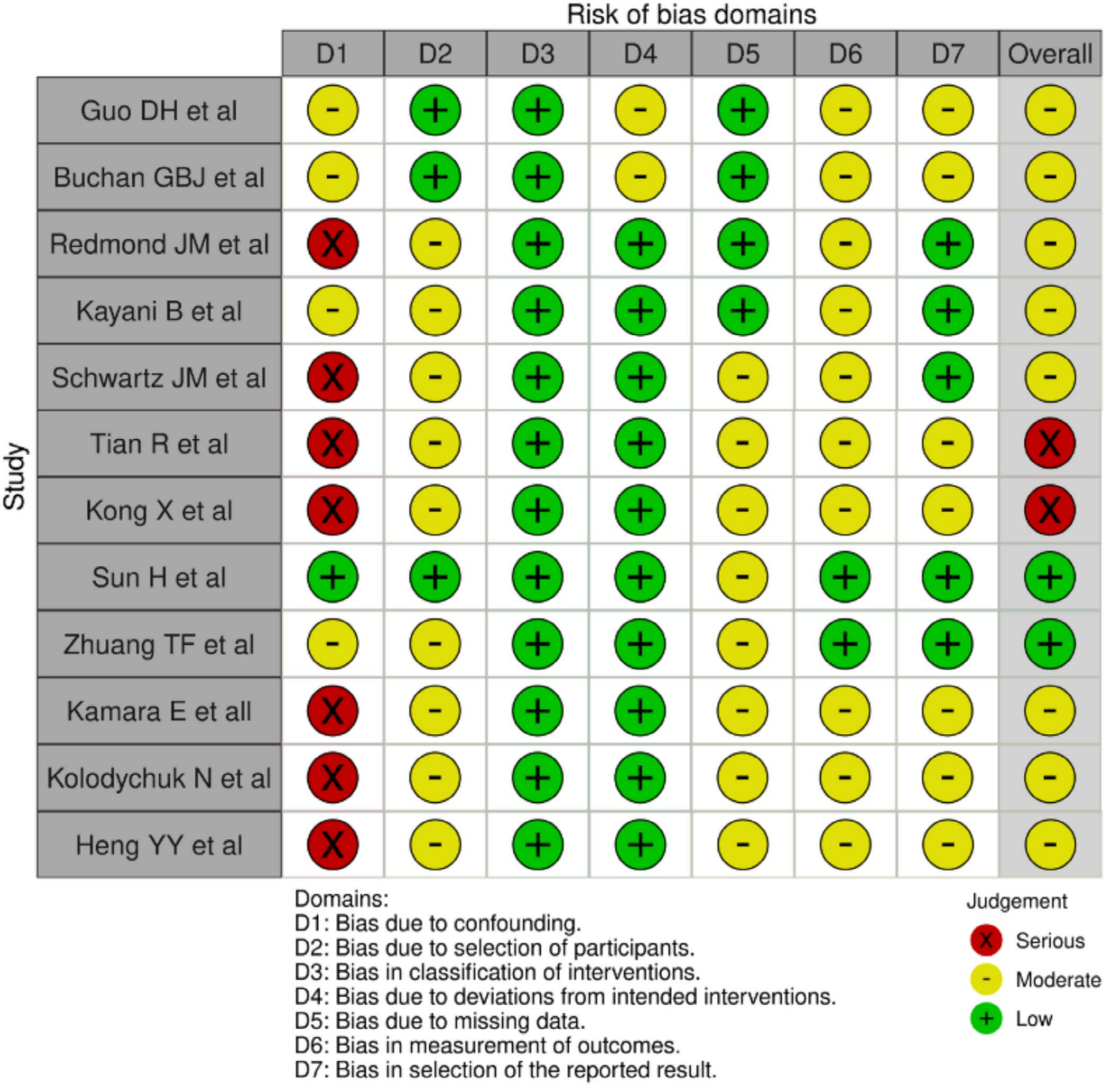
ROBINS‐I risk of bias analysis.

The inclusion and exclusion criteria are summarized in Table 2. Studies were included if they assessed the learning curve associated with robotic total hip arthroplasty (rTHA), defined by factors such as operative time, complication rates, radiographic accuracy, and functional outcomes. Both quantitative and qualitative outcome measures were considered. Exclusion criteria encompassed studies that did not focus on robotic total hip arthroplasty, those evaluating non‐robotic surgical techniques exclusively, or articles lacking measurable outcomes relevant to learning curves or surgical proficiency (Figure 2).

**TABLE 2:** Inclusion criteria.

Criteria	Inclusion	Exclusion
Population	Patients undergoing robotic total hip arthroplasty (rTHA)	Studies not involving robotic total hip arthroplasty
Intervention	Use of robotic‐assisted systems for total hip arthroplasty	Studies focusing solely on conventional or non‐robotic surgical techniques
Outcomes	Studies reporting quantitative or qualitative outcomes (e.g., operative time, complication rates, radiographic accuracy, functional scores)	Articles without measurable outcomes relevant to learning curves or surgical proficiency
Study design	All study designs evaluating the learning curve in robotic total hip arthroplasty	None explicitly excluded based on study design (provided they meet other inclusion criteria)
Language and accessibility	Published in English and accessible in full‐text format	Non‐English studies or inaccessible full‐text articles

### 2.3 Screening, Appraisal and Extraction

All identified studies were uploaded to COVIDENCE for duplicate removal and systematic screening of titles and abstracts. Three independent reviewers (AGK, SSG, SRN) conducted the initial screening. Studies were considered eligible if they were original research articles published in English and reported data on the learning curve in robotic total hip arthroplasty. Full‐text screening was performed for studies meeting the inclusion criteria, with further appraisal conducted by two independent reviewers. Discrepancies were resolved through consensus discussions with MK. Basic demographic information, data pertaining to the surgeons included in the study, and quantitative data on LCs were extracted from each study.

### 2.4 Risk of Bias

Three reviewers independently assessed the methodological quality of the included studies using the Risk of Bias In Non‐randomized Studies of Interventions (ROBINS‐I) tool [29]. This tool was chosen due to its comprehensive evaluation of bias across seven domains, including confounding, participant selection, classification of interventions, deviations from intended interventions, missing data, measurement of outcomes, and selection of the reported result. Discrepancies in assessments were resolved through discussion and consensus, with a fourth reviewer consulted if disagreements persisted.

### 2.5 Weighted Mean and Median

To provide a representative summary of the learning curve metrics across studies, weighted means and medians were calculated where applicable. For continuous variables such as operative time, complication rate, and radiographic deviation, the weighted mean was determined by multiplying each study's outcome by its sample size and dividing the sum by the total number of patients across all included studies. When data were skewed or when the distribution of values varied significantly across studies, the weighted median was reported to better reflect central tendency. These summary measures allowed for clearer comparisons across studies with varying sample sizes and methodologies, particularly in reporting operative efficiency and accuracy during the learning curve of robotic total hip arthroplasty.

## 3 Results

This scoping review screened 409 studies, with 28 full‐text articles meeting the inclusion criteria for further assessment. Ultimately, 12 studies were included. The characteristics of the included studies are detailed in Table 3.

### 3.1 Risk of Bias

The risk of bias analysis in this table shows variability across studies, with 9 studies rated as having “Serious” risk in at least one domain, particularly bias due to confounding. The most frequently observed risk category is “Moderate,” appearing in multiple domains across nearly all studies. Studies such as Sun et al. and Zhuang et al. exhibit lower risk overall, with most domains rated as “Low” or “Moderate.” Conversely, studies like Hecht et al., Tian et al., and Kong et al. have an overall “Serious” risk due to multiple “Serious” ratings across domains, notably in D1. The distribution suggests that while many studies maintain a moderate level of bias, a significant portion has a higher risk in key areas, potentially impacting the reliability of their conclusions.

**TABLE 3:** Basic study characteristics.

Title	Author	Year	Country	Study domains covered	Conclusion
Total hip arthroplasty with robotic arm assistance for precise cup positioning: A case–control study [30]	Guo et al.	2022	China	OT; LL; ACI	The robot‐assisted THA group had a higher proportion of acetabular prostheses in Lewinnek's safety zone and significantly lower LLD compared to the conventional THA group. The MAKO robot improved the accuracy of implant placement in THA.
The learning curve for a novel, fluoroscopy‐based robotic‐assisted total hip arthroplasty system [31]	Buchan et al.	2023	USA	OT; ACI	Adoption of fluoroscopy‐based RA‐THA involves a brief learning curve of 12 cases, with the most significant improvements in surgical efficiency seen during acetabular cup placement.
The learning curve associated with robotic‐assisted total hip arthroplasty [32]	Redmond et al.	2015	USA	OT; LL; ACI	A learning curve was observed in robotic‐assisted total hip arthroplasty, with decreased acetabular component outliers and operative time as experience increased. No learning curve was noted for technical problems or complications. Satisfactory acetabular component positioning and leg length matching were consistently achieved, with few outliers in either category. Robotic‐assisted total hip arthroplasty has a learning curve, with improvements in operative time and component accuracy after 35 cases.
The learning curve of robotic‐arm assisted acetabular cup positioning during total hip arthroplasty [33]	Kayani et al.	2021	UK	OT; LL; ACI	Integration of robotic‐arm assisted acetabular cup positioning during THA was associated with a learning curve of 12 cases for operative times and surgical team confidence levels. However, there was no learning curve effect for accuracy in restoring native hip biomechanics or achieving planned acetabular cup positioning and orientation.
The learning curve for robotic‐assisted total hip arthroplasty in low, medium, and high‐volume surgeons [34]	Schwartz et al.	2024	USA	OT; ACI	High‐volume surgeons have a learning curve of about 20 cases, whereas low‐ and medium‐volume surgeons have a longer learning curve, which was not measurable in this study.
Precise acetabular positioning, discrepancy in leg length, and hip offset using a new seven‐axis robot‐assisted total hip arthroplasty system requires no learning curve: a retrospective study [35]	Tian et al.	2023	China	OT; LL; ACI	The surgical team required a learning curve of 17 cases with the RA‐THA system to become proficient. There was no learning curve for parameters such as LLD, hip offset, or accuracy of acetabular prosthesis positioning. During the proficiency phase, the RA system was superior to conventional THA in controlling leg length and ensuring accurate acetabular cup placement.
A retrospective study comparing a single surgeon's experience on manual versus robot‐assisted total hip arthroplasty after the learning curve of the latter procedure—A cohort study [36]	Kong et al.	2020	China	OT; LL; ACI	In the surgeon's series, proficiency in robot‐assisted THA was achieved after a learning curve of 14 cases. During the proficiency phase, the robot demonstrated superior cup positioning compared to the manual technique.
The learning curve of a novel seven‐axis robot‐assisted total hip arthroplasty system: a randomized controlled trial [37]	Sun et al.	2024	China	OT; LL; ACI	This study demonstrated that the surgical team required a learning curve of 13 cases to become proficient with the robotic THA system. The duration of the operation, total blood loss, and drainage gradually decreased during the learning curve stage, with these differences being statistically significant.
Preliminary study of short‐term outcomes and learning curves of robotic‐assisted THA: comparison between closed platform robotic system and open platform robotic system [38]	Zhuang et al.	2023	China	OT; LL; ACI	The authors found that open and closed robotic‐assisted THA yielded similar surgical outcomes and safe zone outlier rates. Both methods had comparable learning curves and precise acetabular component positioning. The difference in safe zone positioning from the learning to proficiency phase was minimal and not statistically significant. Thus, robotic‐assisted THA is considered an effective method for achieving the planned acetabular cup position.
Adoption of robotic vs. fluoroscopic guidance in total hip arthroplasty: is acetabular positioning improved in the learning curve [39]	Kamara et al.	2017	USA	OT; ACI	Adoption of robotic techniques delivers significant and immediate improvements in the precision of acetabular component positioning during the learning curve. Although fluoroscopy benefits from increased experience, a learning curve exists before significant improvements in precision are achieved.
Can robotic technology mitigate the learning curve of total hip arthroplasty [40]	Kolodychuk et al.	2021	USA	OT; LL	Robotic arm‐assisted technology enabled a newly trained surgeon to achieve results and outcomes comparable to those of experienced surgeons in both anterior and posterior hip arthroplasty.
Conventional vs. robotic arm assisted total hip arthroplasty (THA) surgical time, transfusion rates, length of stay, complications and learning curve [41]	Heng et al.	2018	Australia	OT	The reduction in length of stay, comparable surgical times, and potential for fewer complications may outweigh the increased initial costs associated with the robotic system.

Abbreviations: ACI, acetabular component inclination; LL, leg length; OT, operating time.

**TABLE 4:** Operating times with corresponding learning curves alongside levels of experience of participants.

Study	Operating time (min)	Learning curve (number of cases)	Robot	Surgeon experience
Guo et al.	91.37 ± 17.34	13.0	MAKO	10 years—Conventional
Buchan et al.	38.8 ± 7.1	12.0	ROSA	Attending Orthopedic Surgeon
Redmond et al.	79.8 ± 27.0	35.0	MAKO	
Kayani et al.	57.5 ± 3.7	12.0	MAKO	
Schwartz et al.	70	20.0	MAKO	Board certified Orthopedic Surgeon
Tian et al.	94.43 ± 18.04	17.0	Jianjia seven‐axis robotic arm	Orthopedic Surgeon—No prior robotic experience
Kong et al.	95.92 ± 15.64	14.0	MAKO	Highly experienced in manual THA
Sun et al.	104.26 ± 19.33	13.0	YUANHUA‐THA	Single high volume surgical team
Zhuang et al.	76.7 ± 12.1	9.0	MAKO	1 senior surgeon
Kamara et al.	113		MAKO	Fellowship trained—no prior robotic experience
Kolodychuk et al.	83.6 ± 18.4	12.0	MAKO	Fellowship trained—no prior robotic experience
Heng et al.	96.7 ± 20.1		MAKO	Highly experienced in conventional—no prior robotic experience

### 3.2 Operating Time

This aspect of the scoping review focused on 14 studies examining LC related to operative time, showing evidence that operative time in robotic total hip arthroplasty (THA) generally improves with a surgeon's experience, with the LC overcome between 7 and 35 cases [30, 31, 32, 33, 34, 35, 36, 37, 38, 39, 40, 41, 42, 43]. The weighted mean number of cases required to reach proficiency was 16.4, with a median of 13 cases. Schwartz JM et al. found that low‐volume surgeons (1–15 cases/year) had significantly longer operative times than medium (16–50 cases/year) and high‐volume (51+ cases/year) surgeons, with LC overcome by the 21st case [34]. Definitions of “proficiency” varied across studies. In several cases, proficiency was defined by the point at which operative time plateaued, indicating that additional experience no longer resulted in a significant reduction in surgical duration. Other studies inferred proficiency based on complication rates, surgical volume, or consistency in implant positioning. While Kayani et al. used cumulative summation (CUSUM) analysis to identify the LC at 12 cases, reaching proficiency at 38, with no significant difference in operative times between manual and robotic THA [33]. Findings in more novel robotic systems show no significant LC; others report inflection points around 7–13 cases, with shorter operative times in the proficiency phase [35, 36, 37, 38, 42]. Differences in operative time based on surgeon experience vary between posterior and anterior approaches, but no data is available for varying LCs between the approaches [40, 43] (Table 4).

### 3.3 LLD

The LLD domain explores 9 studies that reference LLD data [30, 32, 33, 35, 36, 37, 38, 40, 43]. LLD is addressed as a secondary outcome in all of these studies, with 2 studies reporting significantly smaller LLD with robotic compared to conventional THA and 1 study showing no significant differences in LLD [30, 35, 37]. Six studies found no significant difference in LLD with robotic THA experience, and different robotic approaches also showed no differences in LLD [32, 33, 35, 36, 38, 40, 43]. Overall, there is little evidence to support the notion of a significant LC pertaining to LLD.

### 3.4 ACI

The ACI domain encompasses 10 studies [30, 31, 32, 33, 34, 35, 36, 37, 38, 39]. Four studies show no significant differences in ACI between robotic and conventional THA ACI, while three studies found significant differences or higher proportions of cases within Lewinnek's zone for robotic THA [30, 31, 33, 35, 36, 37, 39]. However, five studies showed no significant effect of experience on ACI metrics in robotic THA [31, 32, 34, 35, 36, 38]. Overall, the evidence does not support a significant learning curve for ACI with robotic THA.

### 3.5 LC

The weighted mean per n cases was 16.4 with the median being 13 cases based on operating time only. There was limited evidence on learning curves for ACI and LLD. This is summarized in Table 4.

## 4 Discussion

This scoping review contributes to the existing literature by providing a comprehensive synthesis of learning curves associated with robotic total hip arthroplasty, an area that remains underexplored in comparison to conventional THA techniques. Unlike previous studies that primarily focus on overall outcomes, this review systematically evaluates operative time reduction, complication rates, and radiographic accuracy across multiple studies, ultimately finding evidence of a learning curve before surgeons achieve proficiency in terms of operative time. This finding provides a concrete reference point for surgical training programs and informs expectations for early adopters of robotic THA. This is the first scoping review providing an assessment of learning curves using both clinical and radiographic measures, filling a critical gap in the current understanding of robotic‐assisted THA.

### 4.1 Leg Length Discrepancy and Acetabular Component Inclination

Robotic systems offer the potential to enhance the consistency and accuracy of biomechanical execution in orthopedic procedures, particularly THA, where precise component placement is essential for joint function and longevity [10, 44, 45, 46, 47, 48]. LLD and ACI serve as meaningful indicators of procedural and technical success. LLD has a direct impact on postoperative pain, mobility, and patient satisfaction, with discrepancies correlating with lower Oxford Hip Scores and a reported incidence of up to 27% post‐THA [49, 50, 51, 52, 53]. ACI determines the spatial orientation of the acetabular cup and is essential for maintaining joint stability and minimizing complications such as dislocation, impingement, and abnormal wear [49, 50, 51, 54]. In the context of LCs, the accurate restoration of LLD and ACI likely reflects a surgeon's progression toward procedural mastery. Robotic‐assisted THA enhances the reproducibility of these outcomes through tools such as preoperative planning software, intraoperative navigation, and haptic feedback [49, 50, 51]. However, despite the technological advantage, early adopters may still struggle to consistently achieve optimal LLD and ACI during the initial phase of implementation. Variability in these outcomes during early cases may highlight the nuanced technical demands of the procedure, even with robotic assistance. Additionally, the impact of surgical approach, such as anterior versus posterior, on the ability to control these parameters remains poorly understood [52, 53]. Approaches differ in terms of patient positioning, visualization, and intraoperative imaging, which could affect both the learning trajectory and the consistency of radiographic outcomes. Understanding how these factors influence the precision of LLD and ACI over time is essential for interpreting learning curves more holistically and for designing targeted training interventions that prioritize these high‐value clinical endpoints.

### 4.2 Surgeon Specific Factors

Postoperative outcomes, such as early revision rates due to malposition or instability and dislocation rates linked to component accuracy, further contextualize technical success in clinical practice [55]. Surgeon‐specific metrics, including case volume required to achieve proficiency and procedural step completion times, offer an additional perspective on learning curve progression [56]. Standardizing these assessments across future studies will be essential for establishing objective benchmarks and guiding training protocols in robotic‐assisted hip arthroplasty.

Surgeon experience is a critical factor influencing the learning curve in rTHA. In many of the included studies, the operating surgeons were either attending‐level or fellowship‐trained, yet had no prior experience with robotic systems. This is a noteworthy point, as it enhances the validity of the reported learning curves by minimizing confounding from previous robotic exposure. Notably, evidence from general surgery literature suggests that surgeons with greater experience in open procedures tend to demonstrate superior performance during the adoption of robotic techniques, compared to less experienced counterparts [57]. This highlights the importance of foundational surgical expertise in facilitating the transition to robotic platforms. Case complexity plays a critical role in shaping the learning curve for rTHA. In a study examining the direct anterior approach for patients with developmental dysplasia of the hip (DDH), cumulative summation (CUSUM) analysis demonstrated that surgeons required approximately 43 cases to achieve consistent operative proficiency [58]. In contrast, for routine primary THA cases, trainees were able to reach performance levels comparable to senior surgeons after just 10 cases [59]. This stark difference highlights the substantial impact of anatomical complexity on the pace of surgical skill acquisition.

Technical success in rTHA is central to evaluating the associated learning curve. In the included studies, it was generally defined by implant positioning accuracy, restoration of biomechanics, and the ability to complete the procedure without conversion to manual techniques. Objective measures include acetabular cup alignment within ±5° of the preoperative plan, leg length restoration, operative time, fluoroscopy use, and intraoperative re‐registrations. However, variability in outcome reporting limited our ability to analyze these parameters. Complications such as intraoperative fractures, soft tissue injury, and conversions are also important indicators of technical difficulty but were not consistently reported in the studies reviewed [60].

Understanding technical success and its associated learning curve is essential for optimizing training programs in rTHA. By identifying key proficiency markers, such as implant positioning accuracy, operative time reduction, and complication rates, training protocols can be structured to accelerate the learning process while maintaining patient safety [61].

### 4.3 Educational Applications

The establishment of objective benchmarks, including the number of cases required to achieve competency and the reduction in intraoperative errors over time, allows for more tailored surgical education. Additionally, tracking intraoperative metrics such as fluoroscopy time, robotic re‐registration rates, and procedural step efficiency can help guide trainees in recognizing areas requiring improvement [62]. Standardizing these assessments across training centres could enable the development of simulation‐based modules and structured mentorship programs, ensuring a progressive, evidence‐based approach to robotic surgical skill acquisition. The integration of robotics into competency‐based training can ensure all future surgeons are well versed with this skill in the form of EPA (“entrustable professional activities”) assessments—defined as tasks that a trainee can carry out unsupervised once competence has been demonstrated. This fosters greater consistency in surgical performance, ultimately leading to more accurate and reproducible outcomes in operative procedures [63]. Since the integration of the European Working Time Directive, training hours were reduced alongside a significant cut in surgical exposure with trainees performing 15% fewer operations in a trial period of 58 h weeks [64]. However, incorporating the knowledge of LC's and minimum case numbers, training can be optimized around these regulations. Moreover, understanding the variability in learning curves among different robotic platforms and surgeon experience levels can inform individualized training strategies, reducing the risk of early complications and optimizing long‐term surgical outcomes [17]. As robotic technology continues to advance, integrating these findings into competency‐based training frameworks will be critical to ensuring safe and effective adoption of rTHA.

### 4.4 Limitations

Despite providing valuable insights into the learning curve and technical success of rTHA, this scoping review has several limitations. First, the absence of a meta‐analysis limits the ability to quantitatively synthesize findings and assess statistical significance across studies. As a result, conclusions are based on medians and weighted means rather than pooled effect sizes. To address methodological heterogeneity and variation in participant training levels, normality was assumed across datasets, which may have introduced bias. Subgroup analysis by surgeon experience level was not feasible due to insufficient data. Second, although this review attempts to categorize outcomes by robotic system, small sample sizes limit this to an overall value and an overall N the generalizability of these comparisons. Large‐scale, prospective studies across the NHS, particularly involving trainees, are needed to enable standardized comparisons between platforms such as Mako, ROSA, and NAVIO [65, 66, 67]. Additionally, despite a comprehensive search strategy, publication bias or indexing limitations may have led to the omission of relevant studies. Finally, this review focused on three primary metrics as proxies for LCs: implant positioning accuracy, operative efficiency, and intraoperative complications. While informative, these do not fully capture the breadth of robotic surgical proficiency. Important factors such as haptic feedback use, workflow adaptation, and long‐term clinical outcomes were not assessed.

### 4.5 Future Directions

Future studies should incorporate detailed subgroup analyses stratified by surgical training level, including trainees, fellows, and consultant‐grade surgeons, to more accurately characterize the progression of learning curves across varying levels of experience in robotic‐assisted hip arthroplasty. Identifying distinct proficiency benchmarks for each group could facilitate the development of tailored training pathways and clearly defined procedural milestones.

In addition, a deeper understanding of how factors such as prior surgical experience, familiarity with robotic platforms, and case complexity influence the rate and consistency of skill acquisition is critical for informing individualized educational strategies. These findings would support the evolution of competency‐based training frameworks, underpinned by artificial intelligence models capable of integrating surgeon‐specific variables and tracking learning trajectories. Such models could enable dynamic, data‐driven estimates of the number of cases required to achieve procedural proficiency, ultimately contributing to safer adoption, more effective curriculum design, and evidence‐based credentialing within robotic surgical training.

## 5 Conclusions

This scoping review highlights the learning curve associated with rTHA, emphasizing key metrics such as implant positioning accuracy, incidence of LLD, and operating times. While early cases may demonstrate increased operative time and minor deviations from planned component placement, technical success improves as surgeons gain experience, with proficiency typically achieved after a defined number of cases. The findings suggest that standardized assessments of learning progression, including objective benchmarks and intraoperative metrics, could optimize training protocols and enhance patient safety. However, variability in study designs, differences in robotic platforms, and the lack of a meta‐analysis limit the generalizability of these results. Future research should focus on defining proficiency thresholds for different surgical experience levels, conducting direct comparisons between robotic systems, and integrating competency‐based training models to facilitate the safe and efficient adoption of robotic technology in hip arthroplasty.

## Author Contributions

**Abith Ganesh Kamath:** writing – original draft, investigation, conceptualization, methodology, writing – review and editing, visualization, formal analysis. **Saran Singh Gill:** writing – review and editing, writing – original draft, investigation, formal analysis. **Srikar Reddy Namireddy:** investigation, writing – original draft, writing – review and editing, formal analysis. **Matija Krkovic:** supervision.

## Conflicts of Interest

The authors declare no conflicts of interest.
